# Supplementary material for: ViruSurf: an integrated database to investigate viral sequences
Source: Nucleic Acids Res. 2020 Oct 12;49(D1):D817–24. doi: 10.1093/nar/gkaa846 (PMC7778888; doi:10.1093/nar/gkaa846)
Supplement: gkaa846_Supplemental_File [file gkaa846_supplemental_file.pdf]

ViruSurf supplementary data

Arif Canakoglu [1,*], Pietro Pinoli [1,*], Anna Bernasconi [1],
Tommaso Alfonsi [1], Damianos P. Melidis [2] and Stefano Ceri [1†]

[1]Dipartimento di Elettronica, Informazione e Bioingegneria, Politecnico di Milano, Via Ponzio 34/5, 20133 Milano, Italy
[2]L3S Research Center, Leibniz University Hannover, Appelstr. 9a, 30167 Hannover, Germany

[*]Co-first authors
[†]Corresponding author. Tel: +39 02 2399 3532; Fax: +39 02 2399 3411; Email: stefano.ceri@polimi.it

Table S1: Description of Attributes in the Logical schema

Sequence contains virus sequences expressed as strings of nucleotides (encoded in the `nucleotide_sequence` field). `Accession_id` is a unique key used to backtrack the sequence in its original database. Eventually, we also store an `alternative_accession_id`, in case the sequence is also present in a second public database. Sequences belong to a specific `strain_name`; they are either reference or normal sequences (`is_reference`), complete or partial (`is_complete`), thus with a certain `length`; they correspond to a positive or negative `strand` (strictly dependent on the type of virus, that could be single/double strand). We also compute two useful values: the percentage of G and C bases (`gc_percentage`) and of unknown (N) bases (`n_percentage`) throughout the sequence, for quality assessment purposes. When available, we store `lineage` and associated `clade` to which the sequence belongs, as computed by GISAID. As a central table, the Sequence table contains four foreign keys fields to connect to `Virus`, `HostSample`, `SequencingProject` and `ExperimentType` tables.
Virus captures the relevant information of the analyzed species, summarizing the most important levels of the taxonomy branch it belongs to. Specifically, the `taxon_id` – with the corresponding literal specification `taxon_name` – corresponds to the numerical ID of the NCBI Taxonomy (`https://www.ncbi.nlm.nih.gov/taxonomy`) term describing the virus. This belongs to a `species` that derives from a `genus`, which is part of a `sub_family` that in turn is within a `family`. In addition, `equivalent_list` contains an array of alternative terminology for the same species. As to the specific molecule of the virus, we store the type (DNA/RNA) in `molecule_type` and the other structural characteristics in `is_single_stranded` and `is_positive_stranded`.
HostSample represents the biological sample from the host organism. Its NCBI Taxonomy nomenclature is identified by the `host_taxon_id` and related `host_taxon_name`. `Collection_date` captures the temporal information of sequence extraction from an `isolation_source` in an `originating_lab`, which is in a `region`, within a `country`, inside a `geo_group`. In rare cases also the host organism `age` and `gender` are available.
ExperimentType stores information regarding the experiment used to retrieve sequences from biological samples. This regards the `sequencing_technology`, the `assembly_method` and the `coverage` (i.e., number of unique reads that include a specific nucleotide in the reconstructed sequence).
SequencingProject captures information about the organization that stands behind the collected sequence. Usually there is information about a `sequencing_lab` (also referred to as "submitting lab", the `submission_date` (different from the date of collection, captured in the HostSample entity), the `database_source`, indicating the original database from which ViruSurf collects the information, and the `bioproject_id` when available (an external reference to the NCBI BioProject database `https://www.ncbi.nlm.nih.gov/bioproject/`).
Annotation contains information on the structure of the full sequence. Each sub-sequence is defined by a `feature_type` (e.g., gene, protein, coding DNA region, or untranslated region, molecule patterns such as stem loops and so on), coordinates (`start` and `stop`), the containing `gene_name`, the `product` protein. For annotations related to proteins, we store the corresponding pre-computed `nucleotide_sequence`, `aminoacid_sequence`, and an `external_reference` corresponding to NCBI Protein accessions.
NucleotideVariant contains the variants of a specific sequence, computed with respect to the "reference sequence" that is most adopted for the concerned virus species. Characterizing fields include `sequence_original` and `sequence_alternative`, referring respectively to the positions `start_original` and `start_alternative`. `Variant_length` and `variant_type` (e.g., DEL, INS, SUB) complete the identifying information.
VariantImpact includes annotations of each variant about its `effect` (e.g., distruptive, conservative, synonymous) on known genes (`impacted_gene_name`) with a certain `putative_impact` (e.g., LOW, MODERATE, HIGH, MODIFIER).
AminoacidVariant contains the variants w.r.t. an amino acid sequence stored in the specific Annotation. As for the variants of nucleotides, we store the information about the original/alternative sequence, the start position, the length and type of variant.

Table S2: Mappings between variables of ViruSurf and variables of NCBI direct sources (API Nuccore `https://www.ncbi.nlm.nih.gov/books/NBK25497/` and HTML interface `https://www.ncbi.nlm.nih.gov/labs/virus/vssi/#/virus`). For NCBI we specify both the terminology used in the xml/json export files and the one used in their interfaces. In each cell we specify the mapped field, or ✓ if that field is calculated by our pipelines (for example most information about the Virus are computed using the NCBI Taxonomy services), or × when that information is not available. Sometimes we report the double values (e.g., INSDCQualifier/host), this notation indicates that the same information is available under two different elements.

ViruSurf	NCBI Nuccore	NCBI Virus HTML
Sequence.AccessionId	INSDSeq_accession-version	Accession
Sequence.AlternativeAccession	×	×
Sequence.StrainName	INSDQualifier_value/isolate	GenBank_Title
Sequence.IsReference	INSDKeyword	×
Sequence.IsComplete	INSDSeq_definition	Nuc_Completeness
Sequence.Strand	✓	×
Sequence.Length	INSDSeq_length	Length
Sequence.GC%	✓	×
Sequence.N%	✓	×
Sequence.Clade	×	×
Sequence.Lineage	×	×
Virus.TaxonomyID	✓	×
Virus.TaxonomyName	INSDSeq_organism	GenBank_Title
Virus.Species	✓	Species
Virus.Genus	✓	Genus
Virus.Subfamily	✓	×
Virus.Family	✓	Family
Virus.EquivalentList	✓	×
Virus.MoleculeType	INSDSeq_moltype	×
Virus.SingleStranded	INSDSeq_strandedness	×
Virus.PositiveStranded	✓	×
HostSample.TaxonomyID	✓	×
HostSample.TaxonomyName	INSDQualifier_value/host	Host
HostSample.CollectionDate	INSDQualifier_value/collection_date	Collection_Date
HostSample.IsolationSource	INSDQualifier_value/isolation_source	Isolation_Source
HostSample.Originating Lab	×	×
HostSample.GeoGroup	✓	×
HostSample.Country	INSDQualifier_value/country	Geo_Location
HostSample.Region	INSDQualifier_value/country	Geo_Location
HostSample.Age	INSDQualifier_value/host	×
HostSample.Gender	INSDQualifier_value/host	×
ExperimentType.SequencingTechnology	INSDSeq_comment/Sequencing Technology	×
ExperimentType.AssemblyMethod	INSDSeq_comment/Assembly Method	×
ExperimentType.Coverage	INSDSeq_comment/Coverage	×
SequencingProject.SubmissionDate	INSDReference_title/Direct Submission	×
SequencingProject.SequencingLab	INSDReference_title/Direct Submission	×
SequencingProject.DatabaseSource	✓	Sequence_Type
SequencingProject.BioprojectId	INSDXref_id/BioProject	×

Table S3: Mappings between variables of ViruSurf and of COG-UK metadata file, provided in CSV format on their web page (`https://www.cogconsortium.uk/data/`). The notation used is the same as for Table 2.

ViruSurf	COG-UK metadata csv
Sequence.AccessionId	sequence_name
Sequence.AlternativeAccession	×
Sequence.StrainName	sequence_name
Sequence.IsReference	✓
Sequence.IsComplete	✓
Sequence.Strand	✓
Sequence.Length	✓
Sequence.GC%	✓
Sequence.N%	✓
Sequence.Clade	×
Sequence.Lineage	lineage
Virus.TaxonomyID	✓
Virus.TaxonomyName	✓
Virus.Species	✓
Virus.Genus	✓
Virus.Subfamily	✓
Virus.Family	✓
Virus.EquivalentList	✓
Virus.MoleculeType	✓
Virus.SingleStranded	✓
Virus.PositiveStranded	✓
HostSample.TaxonomyID	✓
HostSample.TaxonomyName	✓
HostSample.CollectionDate	sample_date
HostSample.IsolationSource	×
HostSample.Originating Lab	×
HostSample.GeoGroup	✓
HostSample.Country	country
HostSample.Region	adm1
HostSample.Age	×
HostSample.Gender	×
ExperimentType.SequencingTechnology	×
ExperimentType.AssemblyMethod	×
ExperimentType.Coverage	×
SequencingProject.SubmissionDate	×
SequencingProject.SequencingLab	×
SequencingProject.DatabaseSource	✓
SequencingProject.BioprojectId	×

Table S4: Mappings between variables of ViruSurf and of NMDC metadata file, provided in JSON and HTML format on their Web page (http://nmdc.cn/nCov/globalgenesequence/detail/, followed by the sequence id, e.g., “NMDC60013002-07”). The notation used is the same as for Table 2. Sometimes we report the double values (e.g., glength/✓), this notation indicates that when information is missing in the input file, it is computed by our pipeline.

ViruSurf	NMDC json	NMDC HTML
Sequence.AccessionId	seqName	NMDC Accession
Sequence.AlternativeAccession	gisaid	Gisa Id
Sequence.StrainName	isolate	Isolation Strain
Sequence.IsReference	✓	×
Sequence.IsComplete	✓	×
Sequence.Strand	✓	×
Sequence.Length	glength/✓	Length/✓
Sequence.GC%	✓	×
Sequence.N%	✓	×
Sequence.Clade	×	×
Sequence.Lineage	×	×
Virus.TaxonomyID	✓	×
Virus.TaxonomyName	spciesname	Organism
Virus.Species	✓	×
Virus.Genus	✓	×
Virus.Subfamily	✓	×
Virus.Family	✓	×
Virus.EquivalentList	✓	×
Virus.MoleculeType	✓	×
Virus.SingleStranded	✓	×
Virus.PositiveStranded	✓	×
HostSample.TaxonomyID	✓	×
HostSample.TaxonomyName	host	Host
HostSample.CollectionDate	collectionDateFormat	Collection Date
HostSample.IsolationSource	isolationSource	Isolate Name
HostSample.Originating Lab	samplingPlace	Sampling Place
HostSample.GeoGroup	✓/Country	Country
HostSample.Country	country	Country
HostSample.Region	country	Country
HostSample.Age	×	×
HostSample.Gender	×	×
ExperimentType.SequencingTechnology	sequencingMethods	Sequencing Methods
ExperimentType.AssemblyMethod	jointMethods	Joint Methods
ExperimentType.Coverage	×	×
SequencingProject.SubmissionDate	submitDateFormat	×
SequencingProject.SequencingLab	dept	Organization
SequencingProject.DatabaseSource	✓	×
SequencingProject.BioprojectId	×	×

Table S5: Mappings between variables of ViruSurf and of GISAID metadata file, provided in JSON (as an export file prepared for ViruSurf ad-hoc) and HTML format on GISAID portal, via authorized login. The notation used is the same as for Table 2.

ViruSurf	EpiCoV export json	EpiCoV HTML
Sequence.AccessionId	covv_accession_id	Accession ID
Sequence.AlternativeAccession	×	×
Sequence.StrainName	covv_virus_name	Virus name
Sequence.IsReference	is_reference	×
Sequence.IsComplete	is_complete	Complete
Sequence.Strand	covv_strand	×
Sequence.Length	sequence_length	Length
Sequence.GC%	gc_content	×
Sequence.N%	n_content	×
Sequence.Clade	covv_clade	Lineage (GISAID Clade)
Sequence.Lineage	covv_lineage	Lineage (GISAID Clade)
Virus.TaxonomyID	✓	✓
Virus.TaxonomyName	✓	×
Virus.Species	✓	×
Virus.Genus	covv_type	Type
Virus.Subfamily	✓	×
Virus.Family	✓	×
Virus.EquivalentList	✓	×
Virus.MoleculeType	✓	×
Virus.SingleStranded	✓	×
Virus.PositiveStranded	✓	×
HostSample.TaxonomyID	✓	×
HostSample.TaxonomyName	covv_host	Host
HostSample.CollectionDate	covv_collection_date	Collection date
HostSample.IsolationSource	covv_specimen	Specimen source
HostSample.Originating Lab	covv_orig_lab	Originating lab, Address
HostSample.GeoGroup	covv_location	
HostSample.Country	covv_location	
HostSample.Region	covv_location	Location
HostSample.Age	×	Patient age
HostSample.Gender	×	Gender
ExperimentType.SequencingTechnology	×	Sequencing Technology
ExperimentType.AssemblyMethod	×	Assembly method
ExperimentType.Coverage	×	Coverage
SequencingProject.SubmissionDate	covv_subm_date	Submission date
SequencingProject.SequencingLab	covv_subm_lab	Submitting lab, Address
SequencingProject.DatabaseSource	✓	×
SequencingProject.BioprojectId	×	×
